# Adult Bilateral Ureteroceles Presenting with Lower Urinary Tract Symptoms and Acute Urinary Retention

**DOI:** 10.1155/2018/3186060

**Published:** 2018-06-26

**Authors:** Alexis Rompré-Brodeur, Sero Andonian

**Affiliations:** Division of Urology, McGill University Health Center, 1001 Decarie Boulevard, Montreal, QC, Canada H4A 3J1

## Abstract

Ureterocele is a well-known pathologic entity in the pediatric urology population but remains a diagnostic and treatment challenge in the adult population. Adult ureteroceles remain a diagnostic challenge for the adult urologist. Its prevalence is estimated between 1/500 and 1/4000 patients with a wide variety of clinical presentations. We present the case of a 30-year-old female patient who presented with severe lower urinary tract symptoms (LUTS) and acute urinary retention secondary to prolapsing bilateral single-system orthotopic ureteroceles. She was successfully treated with transurethral unroofing of her bilateral ureteroceles and she is currently asymptomatic. This case represents the first reported case of bilateral ureteroceles presenting with severe LUTS and subsequent urinary retention from the prolapse of one of the ureteroceles. We provide a review of the most recent case series of adult ureteroceles and their outcomes. Transurethral unroofing of the ureterocele is a safe and minimally invasive approach for this disease.

## 1. Introduction

Ureterocele is a well-known pathologic entity in the pediatric urology population but remains a diagnostic and treatment challenge in adult population. Its prevalence is estimated between 1/500 and 1/4000 patients with a wide variety of clinical presentations described [1]. We present a case of a 30-year-old female patient with bilateral single-system ureteroceles and secondary urinary retention successfully treated endoscopically. Her case constitutes the first reported adult case of bilateral ureteroceles causing bladder outlet obstruction and urinary retention.

## 2. Case Presentation

A 30-year-old female was referred to the urology clinic for severe lower urinary tract symptoms (LUTS) in addition to suspicion of a urethral mass. Her symptoms started two years earlier after the indwelling urethral catheter was removed following her Caesarian section. She noticed that a vestibular mass protruded at the level of her urethra and she reported increasing need to strain to void. In addition, this vestibular mass was very painful, and it required manual reduction to allow for her to void. LUTS got progressively worse over the course of the two years. In addition to the dysuria, she had intermittent hematuria, frequency of every hour as well as nocturia 3 to 4 times per night. Her personal past medical history was only positive for a remote appendectomy and a caesarean section. The patient had been previously worked up by her obstetrician since the LUTS appeared after her caesarean delivery. She had repeated urine analyses and cultures that were negative. Abdominal ultrasonography demonstrated the presence of two cystic lesions in the bladder of 2.5cm and 9mm in diameter. The kidneys did not show any hydronephrosis nor hydroureter. Her blood work, including renal function, was unremarkable.

**Figure 1:**
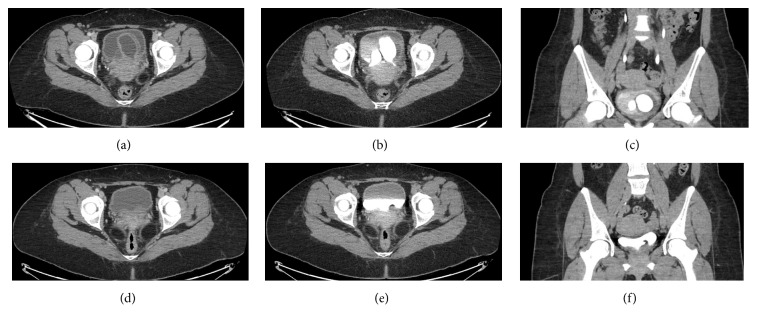
Preoperative (a, b, c) and postoperative (d, e, f) comparison of computed tomography (CT) scans. Preoperative triphasic CT scan images. (a) Contrast-infused axial images of the pelvis demonstrating two large bilateral intravesical cavities. (b) Axial delayed-phase images of the pelvis confirming the diagnosis of bilateral ureteroceles. (c) Coronal delayed-phase images of the pelvis provide another view of the bilateral ureteroceles. Postoperative triphasic CT scan images. (d) Contrast-infused axial images of the pelvis demonstrating the absence of intravesical cavities. (e) Axial delayed-phase images of the pelvis confirming the absence of any residual ureteroceles. (f) Coronal delayed-phase images of the pelvis.

**Figure 2:**
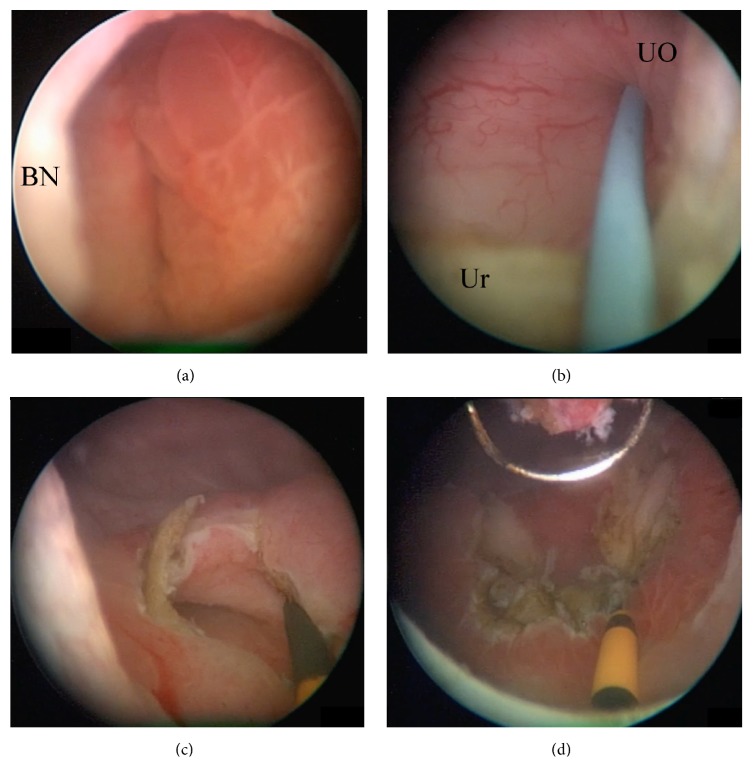
Endoscopic images during transurethral resection of bilateral ureteroceles. (a) View from the bladder neck of the left ureterocele with bullous edematous mucosa that was prolapsing. (b) After unroofing of the left ureterocele, the internal left ureteral orifice was cannulated with a hybrid nitinol-PTFE guidewire. (c) A ureteral catheter was placed over the guidewire. (d) Image of the left bladder trigone after complete resection of the left ureterocele.** BN:** bladder neck.** UO:** ureteric orifice.** Ur:** ureterocele wall.

Examination in lithotomy position revealed a very sensitive erythematous mucosa protruding from the urethra. Patient had to manually reduce the vestibular mass in the sitting position so that she could undergo cystoscopic examination under local anesthesia. Otherwise, it was too painful for the patient. Once reduced, cystoscopy was performed and demonstrated the presence of two large ureteroceles. The left ureterocele, which was significantly larger than the right side, demonstrated erythematous and edematous mucosa indicating that the vestibular mass previously noted by the patient and on examination was likely the wall of the left ureterocele. To rule out duplex system, a triphasic CT-urogram was ordered and it confirmed the patient's bilateral single-system ureteroceles (Figures 1(a)–1(c)). No urolithiasis was identified in both collecting systems. She was offered transurethral “unroofing” of her ureteroceles with placement of bilateral indwelling ureteral stents to reduce risk of ureteral obstruction postoperatively (Figure 2). While the patient was waiting for her elective surgery, she presented to the emergency department in acute urinary retention in addition to significant lower abdominal pain. An indwelling urethral catheter was placed to reduce the prolapsed ureterocele, decompress the bladder, and control her severe pain. Subsequently, she underwent the planned endoscopic procedure. We started with the larger and more symptomatic left ureterocele. As soon as we unroofed the ureterocele, we identified the normal ureteral orifice and placed ureteral catheter over a guidewire (Figure 2). Ureteral catheters were used to avoid injuring the back wall of the ureterocele and avoid injuring the true ureteral orifice within the ureterocele. The same procedure was repeated for the smaller right ureterocele. Once the anterior wall of the ureterocele was resected, we placed indwelling ureteral stents bilaterally. Final pathology demonstrated benign urothelium with cystitis cystica and glandularis in addition to Von Brunn's nests. Indwelling ureteral stents were removed shortly after her operation. At 3-month follow-up, the patient was completely asymptomatic with normal voiding patterns, a normal flow study and renal function. Repeat CT-urogram demonstrated resolution of her bilateral ureteroceles without any signs of ureteral stricture, nor hydronephrosis (Figures 1(d)–1(f)). A voiding cystourethrogram was not performed given that the patient remained asymptomatic at 6 months postoperatively without any signs of vesico-ureteral reflux or urinary tract infections.

## 3. Discussion

A ureterocele is a well-known entity among pediatric urologic population but it remains a challenge in the adult population. Most of our knowledge is based on case reports and case series. Based on autopsy studies, its prevalence in the adult population ranges between 1/500 and 1/4000 [1]. Whereas pediatric ureteroceles are often associated with duplicated collecting systems in nonorthotopic positions, adult ureteroceles are mainly reported in unilateral single systems in intravesical orthotopic positions [1, 2]. The mean age of presentation ranges from the third to the fifth decade (Table 1). Presenting symptoms vary greatly but urinary tract infections remain the most common [2]. Urinary retention secondary to prolapsing ureteroceles in adults has been reported in only seven cases. All of these occurred with unilateral prolapse of a single-system ureterocele [3–9]. This case constitutes the first reported adult case of a bilateral ureteroceles causing bladder outlet obstruction secondary to prolapse of one of the ureteroceles. Similar to pediatric populations, adult ureteroceles can contain stones at a rate of 4–39%. The main composition of theses stones is calcium-oxalate and calcium-phosphate [10]. In the present case, CT scan confirmed the absence of urolithiasis.

Treatment options vary but most reported cases seem to favor a low transverse incision with Collin's knife in a “smiling” fashion, similarly to what is being advocated for the pediatric population (Table 1) [11]. Recently, Holium and KTP lasers have been used to make the incision [12, 13]. Other approaches such as simple puncture or endoscopic unroofing with the resectoscope have also been described (Table 1). Case series have reported rate of developing postoperative vesicoureteral reflux (VUR) to range from 0% to 33% in patients with low transverse “smiling” incision; most cases resolved spontaneously at 6 months of follow-up (Table 1) [14]. Our review of the current literature showed a low (7.1%) risk of postoperative VUR after endoscopic treatment with documented spontaneous resolution in half of the cases with none requiring further treatment, hence reinforcing the widespread clinical practice to investigate adult VUR only in symptomatic patients (Table 1). In our patient's case, we opted for a transurethral unroofing technique as the ureterocele had been prolapsing and causing urinary retention. A “smiling” incision was not performed since excess remnant tissue associated with the low transverse “smiling” incision has been reported to prolapse, requiring a second procedure [15]. Therefore, in our case, the patient was cured with a single transurethral unroofing of both ureteroceles. She did not complain of any VUR symptoms. To our knowledge, this case represents the first reported case of bilateral ureteroceles presenting with severe LUTS and subsequent urinary retention from the prolapse of one of the ureteroceles. At any age of presentation, ureteroceles can be part of a complex malformation of the upper urinary tract and careful investigation is mandatory for an appropriate treatment plan. Transurethral unroofing of the ureterocele is a safe and minimally invasive approach for this disease in adults.

## Figures and Tables

**Table 1:** Review of literature of adult ureteroceles.

**Authors**	**Publication year**	**Mean age/Range**	**Number of cases**	**Bilateral**	**Urinary retention**	**Urolithiasis**	**Method of resection**	**F/U imaging**	**Complication**
**Jimenez et al. [8]**	1976	31	1	0/1	1/1	0/1	Transcutaneous puncture	N/A	N/A
**Sehn et al. [7]**	1981	32	1	0/1	1/1	0/1	Transvesical excision and reimplantation	N/A	0/1
**Sandhu et al. [9]**	1992	N/A	1	0/1	1/1	N/A	N/A	N/A	N/A
**Sekine et al. [3]**	1996	40	1	0/1	1/1	0/1	Transverse incision Collin's knife	IVU, VCUG	0/1
**Chtourou et al. [13]**	2002	48.3	20	4/20	N/S	20/20	Transverse incision Collin's knife	VCUG	1/20 with resolution at 6 months' follow-up
**Spatafora et al. [14]**	2006	18-62	15	4/15	0/15	N/S	Transurethral transverse incision and percutaneous combined approach	VCUG	2/15 low grade VUR
**Shah et al. [9]**	2008	35	16	2/16	N/S	16/16	Transverse incision Holium laser	U/S, IVU, VCUG	3/16 low-grade VUR at 3months, with resolution at 6 months
**Seibold et al. [16]**	2010	48	8	1/8	0/8	5/8	Bugbee wire elec-trode	U/S, VCUG	0/8
**Vijay et al. [11]**	2011	25	26	2/26	0/26	3/26	Transverse incision Collin's knife	U/S, IVU and VCUG	2/26 Low grade VUR asymptomatic
**Isen et al. [15]**	2012	47	5	/05	0/5	2/5	Nephroscopic scissors	VCUG	0/5
**Westesson et al. [4]**	2013	41	1	0/1	1/1	0/1	Transverse incision	Nil	0/1
**Sinha et al. [5]**	2014	35	1	0/1	1/1	1/1	Transurethral unroofing resection	N/A	0/1
**Liu et al. [10]**	2015	31	30	2/30	0/30	2/30	Transverse incision KTP Laser	U/S, IVU, VCUG	1/30 grade I VUR with resolution at 6 months
**Villagomez-Camargo et al. [6]**	2015	24	1	0/1	1/1	0/1	Cohen's reimplantation	Nil	0/1
**Total**	-	-	127	18/127	7/91	52/111	-	-	9/127 low grade VUR. 5/9 had documented spontaneous resolution of VUR at 6 months.

## Abbreviations

BN: Bladder neck
LUTS: Lower urinary tract symptoms
UO: Ureteric orifice
Ur: Ureterocele wall
VUR: Vesicoureteral reflux.
